# Adolescent Binge Drinking Leads to Changes in Alcohol Drinking, Anxiety, and Amygdalar Corticotropin Releasing Factor Cells in Adulthood in Male Rats

**DOI:** 10.1371/journal.pone.0031466

**Published:** 2012-02-08

**Authors:** Nicholas W. Gilpin, Chrisanthi A. Karanikas, Heather N. Richardson

**Affiliations:** 1 Department of Physiology, Louisiana State University Health Sciences Center, New Orleans, Louisiana, United States of America; 2 Department of Psychology (Neuroscience and Developmental Divisions), Neuroscience and Behavior Program, Center for Neuroendocrine Studies, The University of Massachusetts-Amherst, Amherst, Massachusetts, United States of America; University of Groningen, Netherlands

## Abstract

Heavy episodic drinking early in adolescence is associated with increased risk of addiction and other stress-related disorders later in life. This suggests that adolescent alcohol abuse is an early marker of innate vulnerability and/or binge exposure impacts the developing brain to increase vulnerability to these disorders in adulthood. Animal models are ideal for clarifying the relationship between adolescent and adult alcohol abuse, but we show that methods of involuntary alcohol exposure are not effective. We describe an operant model that uses multiple bouts of intermittent access to sweetened alcohol to elicit *voluntary* binge alcohol drinking early in adolescence (∼postnatal days 28–42) in genetically heterogeneous male Wistar rats. We next examined the effects of adolescent binge drinking on alcohol drinking and anxiety-like behavior in dependent and non-dependent adult rats, and counted corticotropin-releasing factor (CRF) cell in the lateral portion of the central amygdala (CeA), a region that contributes to regulation of anxiety- and alcohol-related behaviors. Adolescent binge drinking did not alter alcohol drinking under baseline drinking conditions in adulthood. However, alcohol-dependent and non-dependent adult rats with a history of adolescent alcohol binge drinking did exhibit increased alcohol drinking when access to alcohol was intermittent. Adult rats that binged alcohol during adolescence exhibited increased exploration on the open arms of the elevated plus maze (possibly indicating either decreased anxiety or increased impulsivity), an effect that was reversed by a history of alcohol dependence during adulthood. Finally, CRF cell counts were reduced in the lateral CeA of rats with adolescent alcohol binge history, suggesting semi-permanent changes in the limbic stress peptide system with this treatment. These data suggest that voluntary binge drinking during early adolescence produces long-lasting neural and behavioral effects with implications for anxiety and alcohol use disorders.

## Introduction

Binge drinking is defined by the National Institute on Alcohol Abuse and Alcoholism (NIAAA) as heavy episodic drinking that brings a person's blood alcohol levels to 0.08 gram% (g%, g/dL) or higher (∼4 drinks in women, ∼5 drinks in men within a 2-hour period [1], [2]). This type of drinking is highly prevalent in teenagers [3], [4], possibly due to the high sugar content and packaging of alcoholic beverages (“alcopops” [4]). Adolescent binge drinking has been linked to stress, addiction, and mental health problems throughout the lifespan [5]–[8]. In fact, early onset of heavy drinking is one of the strongest predictors of a lifetime prevalence of alcohol dependence, with children who start drinking at age 14 and younger being four times more likely to become alcohol dependent than those who began drinking at age 20 and older [9]–[15].

The epidemiological literature in humans suggests several possible links between adolescent binge alcohol drinking and negative mental health outcomes such as alcoholism during adulthood. The first possibility is that individuals who binge drink at this early age already have a predisposition to engage in excessive, risky drinking behaviors in adulthood independent of their prior experience with alcohol during adolescence (i.e., binge drinking is an early sign of vulnerability); the second is that adolescent binge drinking results in alcohol exposure (specific pattern of intake, accumulated amount consumed, etc.) that causes long-term neurochemical or neuroanatomical changes within addiction-related brain circuits to increase the risk of alcohol dependence in adulthood; and the third is that both factors (exposure and predisposition) contribute to increased risk of addiction in adulthood. Epidemiological studies suggest increased dependence risk in individuals who begin drinking early in adolescence may be due, at least in part, to the level of alcohol exposure itself, although it is challenging to prove a causal role of adolescent binge alcohol exposure on vulnerability to dependence and related mental health issues in humans [8], [12].

Animal studies provide some support for the correlative findings observed in humans. Adolescent rats and mice engage in higher levels of drinking compared to adults [16]–[19]. In addition, high alcohol consumption early in adolescence correlates with high alcohol consumption later in adolescence [20] and in adulthood [16], [17], [19]. A recent study used scheduled fluid availability [17] in an inbred alcohol-preferring strain of mice to elicit binge-like consumption of alcohol and found that baseline drinking and alcohol preference was higher in animals that began drinking as adolescents compared to animals that began drinking as adults [16].

In the present study, we developed an operant self-administration model to investigate the impact of adolescent binge drinking on vulnerability to heavy drinking and dependence in adulthood. This work was done in male Wistar rats, an outbred strain without a predisposition to addiction. The operant approach was used in part because *involuntary* exposure to binge-like alcohol via systemic injections (but not saline injections or voluntary alcohol drinking) caused long lasting *reductions* in voluntary drinking of alcohol in adulthood (data shown herein). The model also aimed to better mimic the binge alcohol intake observed in many teenagers: heavy, episodic, *voluntary* oral consumption of sweetened alcoholic beverages [4]. The binge drinking exposure period was timed to occur during the first “half” of adolescence (28–42 days of age) to coincide with the pubertal maturation phase [21] of the ∼4-week long developmental period in rodents [21]–[23]. This developmental period in rats is comparable to the age at which the onset of alcohol drinking is associated with the greatest risk for developing alcohol dependence in adulthood in humans (14 years old and under; [3], [13], [24], [25]).

In this study, chronic intermittent alcohol vapor inhalation was used to produce mild physical dependence [26] but robust motivational/emotional dependence (reviewed in [27]). This dependence model elicits an acute withdrawal syndrome similar to what is observed in many human alcoholics: affective disturbances [28], attenuated function of the hypothalamic pituitary adrenal axis [26], and relapse to excessive drinking when alcohol is made available [29], which is sufficient to produce binge-like blood alcohol levels [26], [30]. The present study investigated whether adolescent binge alcohol drinking impacted adult drinking before and after dependence (via chronic intermittent alcohol vapor), as well as passive anxiety-like behavior on the elevated plus maze one month into abstinence from chronic alcohol.

After behavioral testing was completed, corticotropin-releasing factor (CRF) cells were immunolabeled and counted in the lateral portion of the central amygdala (CeA). The CeA is an inhibitory (i.e., GABAergic) structure with lateral and medial subdivisions. The lateral CeA sends dense GABAergic projections to the medial CeA where high quantities of peptides, including CRF, are released (reviewed in [31]). The medial aspect of the CeA is the major output region of the amygdala and sends dense GABAergic projections to downstream effector regions responsible for producing behavioral responses to stress- and alcohol-related stimuli [31]. We counted CRF cells in the CeA because these peptide-producing cells increase in number and mRNA production just prior to adolescence [32], [33], and also because CRF and its receptors have been linked to binge drinking and alcohol dependence [5], [9], [34]–[38].

## Results

### Involuntary binge alcohol exposure during early adolescence reduces baseline drinking in adulthood

**Figure 1:**
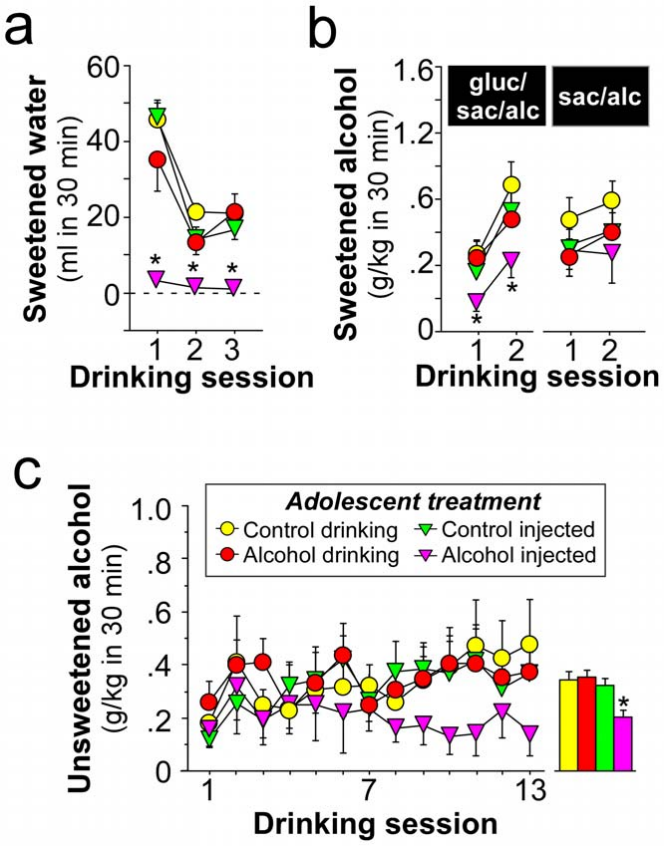
Involuntary alcohol exposure during adolescence reduces baseline drinking in adulthood. Adult drinking data from male Wistar rats previously exposed to involuntary alcohol (“alcohol injected, 2 g/kg *ip*) or voluntary alcohol drinking (four 30-min bouts of home cage drinking of sweetened 5% w/v alcohol) early in adolescence (postnatal days 27, 30, 33, 36, 39). Control animals either received saline injections or voluntary sweetened water drinking on the same adolescent treatment days. Previous exposure to involuntary binge-like alcohol during adolescence via *ip* injections (but not control injections or voluntary alcohol drinking) significantly reduced drinking of sweetened water (a), 5% w/v alcohol sweetened with 3% glucose/0.125% saccharin (left graph, b) but not when the glucose was removed (right graph, b), and 5% w/v unsweetened alcohol (c) in adulthood. Data are expressed as mean ± SEM (n = 8/group). *p<0.05 relative to all control groups.

**Figure 2:**
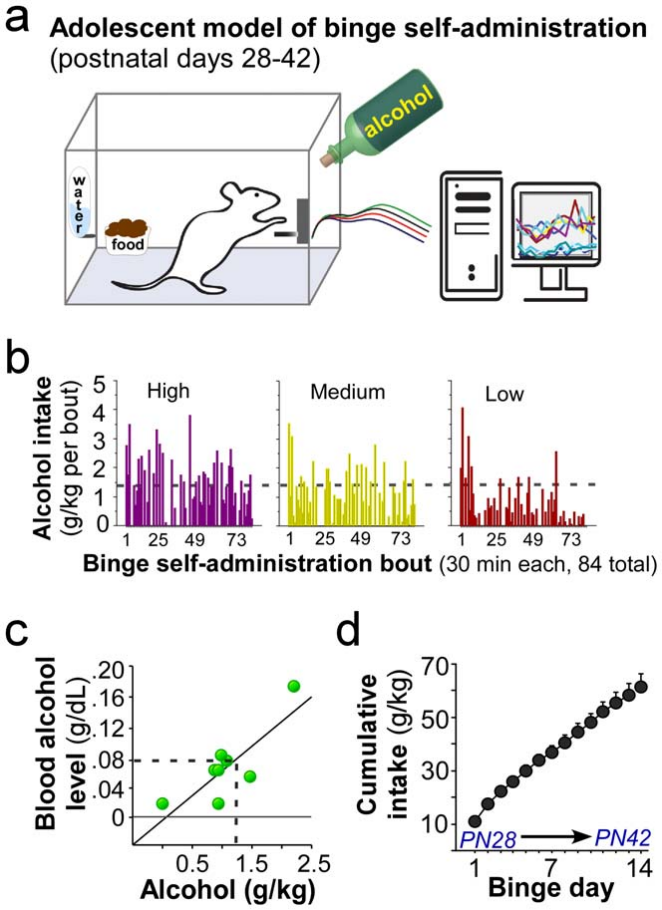
Operant model of binge alcohol self-administration in adolescent male Wistar rats. (a) Illustration of the binge self-administration apparatus. (b) The pattern of alcohol intake (g/kg) from representative high (*n* = 8; 76.8±3.5 g/kg total intake; 27.1±2.1 total number of binges), medium (*n* = 6; 63.6±3.3 g/kg total intake; 22.5±2.7 total number of binges), and low (*n* = 6; 35.3±2.8 g/kg total intake; 35.3±2.8 total number of binges) binging rats over the two week treatment period (14 overnight sessions×6 bouts = 84 sessions total). (c) A scatter plot showing the relationship between g/kg intake and resultant blood alcohol levels in a single bout on the last binge day in a subset of rats. A linear fit trend line is used to estimate the average g/kg intake that results in ≥0.08 g% blood alcohol levels (i.e., ∼1.25 g/kg would be classified as “binge drinking”). As shown in (b) and (c) not all animals binge within a single session and instead vary regarding the bout(s) in which they binge within each overnight session and the total number of binges over the two-week treatment period. (d) By the time animals reach postnatal day (PN) 42 the cumulative intake approaches 50–60 g/kg. Data are expressed as mean ± SEM (c: n = 8 binge rats; d: n = 20 binge rats).

Experimenter-administered (involuntary) alcohol delivery has been useful for studying how different doses of alcohol affect the brain and behavior. During the development of an adolescent binge alcohol model, we first investigated the effects of voluntary vs. involuntary exposure to alcohol in early adolescence (postnatal days 27, 30, 33, 36, and 39) on drinking in adulthood (tests began on postnatal day 72) in male Wistar rats. Alcohol-injected rats were administered a single intraperitoneal (*ip*) injection of 2.0 g/kg of alcohol immediately after the final of four 30-min bouts of access to sweetened water on each of the adolescent treatment days. Voluntary sweetened alcohol intake (home cage drinking) ranged from 0.0 to 2.1 g/kg of alcohol in a single 30-min bout, and the average total intake was 1.7±0.5 g/kg per day. Thus, the two alcohol groups were exposed to comparable levels of alcohol in a single day, although the method and pattern of alcohol delivery differed. Blood-alcohol levels (BALs) in voluntary alcohol drinking rats ranged from 0.0 to 0.1 g/dL. BALs in alcohol-injected rats ranged from 0.10 to 0.20 g/dL (averaged 0.17±0.02 g/dL). **Fig. 1** shows drinking behavior in adulthood from the four adolescent treatment conditions: 1) “Control drinking” (sweetened water), 2) “Alcohol drinking” (sweetened 5% w/v alcohol, ∼1.7 g/kg daily intake), 3) “Control injected” (saline, *ip*) paired with sweetened water drinking, and 4) “Alcohol injected” (2.0 g/kg of alcohol, *ip*) paired with sweetened water drinking. In adulthood, all animals were tested for consumption levels of an experimental solution vs. tap water in daily two-bottle choice home cage drinking tests. Adult drinking data are shown for the following solutions: 1) sweetened water (3% glucose/0.125% saccharin for first three days of testing, **Fig. 1a**), 2) sweetened 10% w/v alcohol (sweetened with 3% glucose/0.125% saccharin for the first two days of testing followed by 0.125% saccharin sweetened alcohol for the next two days, **Fig. 1b**, left and right graphs respectively), and 3) unsweetened 10% w/v alcohol (last 13 days of testing, **Fig. 1c**). Previous exposure to involuntary binge-like alcohol during adolescence via *ip* injections (but not control injections or voluntary alcohol drinking) significantly reduced drinking of sweetened solution in adulthood (effect of adolescent treatment on sweetened water consumption in adulthood; (F(3,84) = 32.20, p<.0001; with *post-hoc* analyses indicating the alcohol injected group had reduced consumption compared to the alcohol drinking and saline injected groups, p<.0001). Adolescent treatment also affected consumption of glucose/saccharin-sweetened alcohol in adulthood (F(3,56) = 3.42, p = .02, with *post-hoc* analyses indicating the alcohol-injected group had reduced consumption compared to the saline-injected and voluntary alcohol drinking groups, p<0.05, *, **Fig. 1b**, left graph). Saccharin-sweetened alcohol consumption was not different between the groups. Finally, adolescent treatment affected unsweetened alcohol (10% w/v) consumption in adulthood (F(3,364) = 5.67, p = .0008, with *post-hoc* analyses indicating that alcohol-injected rats consumed less alcohol compared to saline-injected rats, p<.004, and voluntary alcohol drinking rats, p<0.01, *, **Fig. 1c**).

### Operant model of voluntary binge drinking in adolescent rats

We circumvented the need for involuntary passive delivery of alcohol by developing an operant self-administration model of binge drinking in adolescent rats (**Fig. 2**). Following a short training period (details in methods) adolescent male Wistar rats underwent overnight voluntary binge sessions (six 30-min bouts per night, separated by 90-min time-out periods where the lever was retracted). Animals either had access to sweetened alcohol (10%w/v ethanol made with 3% glucose/0.125% saccharin sweetened water, “Binge”) or sweetened water (responses were capped to equal the sugar intake of the Binge rats, “Control”) in the presence of *ad libitum* food and water during early adolescence (postnatal days 28–42). **Fig. 2b** shows the pattern of alcohol intake (estimated by lever responses) over the two-week period (84 sessions) from representative high (*n* = 8; 76.8±3.5 g/kg total intake; 27.1±2.1 total number of binges), medium (*n* = 6; 63.6±3.3 g/kg total intake; 22.5±2.7 total number of binges, which were defined as 1.25 g/kg or higher alcohol intake as described below), and low (*n* = 6; 35.3±2.8 g/kg total intake; 9.5±0.9 total number of binges) binging rats. **Fig. 2c** shows a scatter plot of the simple regression analysis for a single bout on PN42, indicating that approximately 1.25 g/kg intake results in binge-like (≥0.08 g%) blood alcohol levels. It should be noted that not all animals binge within a single session and instead vary regarding the bout(s) in which they binge (as estimated by ≥1.25 g/kg intake) within each overnight session and the total number of binges over the two-week treatment period. Furthermore, 1.25 g/kg is only an estimate of the amount of alcohol self-administered that would produce ≥0.08 g% blood alcohol levels within a bout, and may be an overestimate in some cases and an underestimate in other cases. Importantly, as is evident in **Fig. 2 (b, d)**, this model elicits episodic drinking patterns and high cumulative voluntary alcohol intake (∼50–60 g/kg) over the short course of early adolescence in outbred rats *without* a predisposition to binge drinking or dependence.

### Effect of adolescent binge drinking on baseline drinking in adulthood

**Figure 3:**
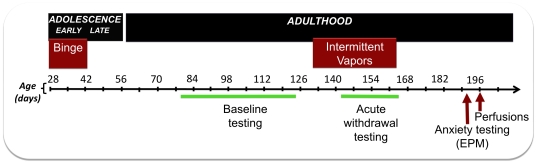
Illustration of the timeline of treatment, behavioral measures, and brain collection for the voluntary binge drinking experiments in male Wistar rats. Voluntary binge alcohol self-administration took place during early adolescence (postnatal days 28–42) and dependence induction (chronic exposure to intermittent alcohol vapors) took place after baseline drinking testing in adulthood. In adulthood, drinking behavior was measured in 30-min operant self-administration tests before and during dependence induction by intermittent vapors. Anxiety-like behavior was measured in the elevated plus maze in abstinence (approximately one month after removal from intermittent alcohol vapors). Brains were collected for CRF immunoreactivity one week after testing for anxiety-like behavior.

**Figure 4:**
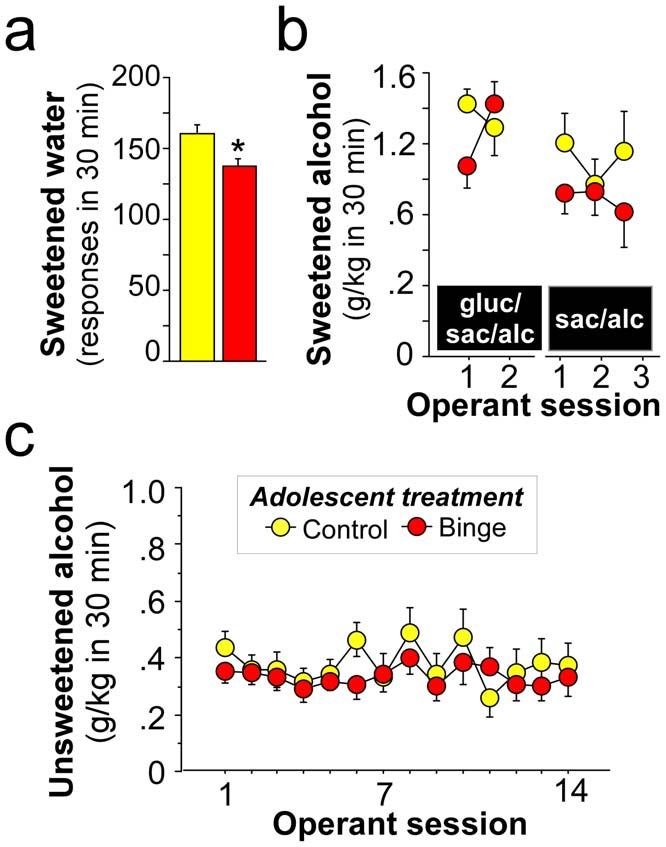
Voluntary binge drinking early in adolescence does not affect baseline drinking in adult male Wistar rats. (a) Responses for sweetened water (3% glucose/0.125% saccharin, 1 day), (b) sweetened 10% w/v alcohol intake (g/kg) with (3 days, left graph) and without (2 days, right graph) glucose, and (c) unsweetened 10% w/v alcohol intake (g/kg, 14 days). Daily operant self-administration tests lasted 30-min in adult rats with an adolescent history of voluntary binge alcohol (red) or control (yellow) drinking alcohol during early adolescence. Data are expressed as mean ± SEM (n = 15–20/group). *p<0.05 relative to rats without a history of adolescent binge alcohol drinking.

**Fig. 3** shows the timeline of treatment, behavioral measures, and brain collection for the voluntary binge drinking experiments. After adolescent treatment, rats were left in the home cage for one month and then tested for self-administration behavior in adulthood (beginning on postnatal day 78). **Fig. 4** shows baseline operant self-administration of 1) sweetened water (3% glucose/0.125% saccharin/water, **Fig. 4a**), 2) sweetened alcohol (two days of 3% glucose/0.125% saccharin/10% w/v alcohol, left graph, **Fig. 4b**; three days of 0.125% saccharin/10% w/v alcohol, right graph, **Fig. 4b**), and 3) thirteen days of unsweetened alcohol (10% w/v alcohol, **Fig. 4c**) self-administration in adulthood. Testing occurred in 30 min sessions approximately five days per week, followed by two days off in the order stated above. Alcohol consumption was normalized to individual body weights and expressed as g/kg intake. Importantly, adolescent binge history did not affect body weight in adulthood (data not shown). Animals with an adolescent history of drinking sweetened water (Control) self-administered significantly more sweetened water in adulthood compared to animals with a history of binge alcohol (F(1,31) = 6.54, *, p = .02, **Fig. 4a**). However, Control and Binge animals exhibited similar baseline self-administration of 10% w/v alcohol whether it was sweetened with glucose and saccharin (**Fig. 4b**, left graph, p>0.05), sweetened with saccharin (**Fig. 4b**, right graph, p>0.05), or unsweetened (**Fig. 4c**, p>0.05). Subsequent measures of baseline home cage drinking was also similar among the groups (data not shown).

### Effect of adolescent binge drinking on dependence-induced alcohol drinking

**Figure 5:**
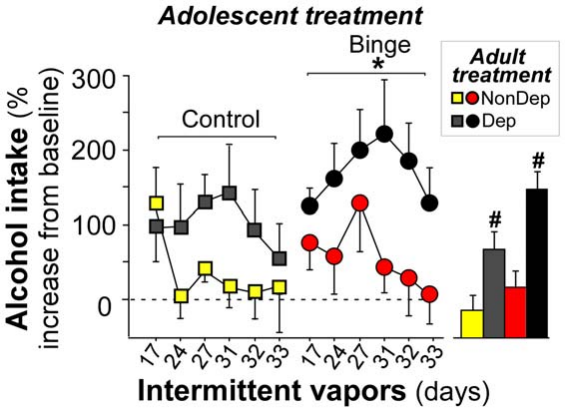
Voluntary binge drinking early in adolescence augments relapse-like drinking in adult non-dependent and dependent male Wistar rats. Chronic intermittent alcohol vapor produced significant elevations in alcohol self-administration by Dependent rats (black) during 30-min operant tests relative to Non-dependent rats. Animals with a history of adolescent binge alcohol drinking (right panel of line graph) also drank more than non-binge controls during adulthood. Data are expressed as mean ± SEM (n = 6–10/group). Dependent and non-dependent groups did not differ in adolescent consumption levels (p = 0.34). *p<0.05 main effect of adolescent alcohol binge drinking. #p<0.05 main effects of adult alcohol dependence.

Rats were next tested for sensitivity to dependence-induced elevations in drinking. Alcohol intake varied across the post-vapor testing period, with g/kg consumption averaging 0.65±0.24 g/kg in Control Dep rats and 0.77±0.15 g/kg in Binge Dep rats overall. **Fig. 5** shows alcohol intake during intermittent (14 hours daily) self-administration tests across weeks 2–5 of chronic intermittent alcohol vapor (“Dependent” or “Dep”) or ambient air (“Non-Dependent” or “NonDep”) in animals that binge drank sweetened alcohol (“Binge”) or sweetened water (“Control”) during early adolescence. To establish whether adolescent binge drinking augmented dependence-induced increases in alcohol intake, drinking levels were normalized to baseline (% increase in g/kg alcohol intake from baseline levels). Alcohol self-administration of 10% w/v alcohol was measured in 30-min tests, 6–8 hours into withdrawal as described previously [26]. This period of withdrawal was chosen because it is a time when dependent animals display mild somatic signs of dependence [26], but robust emotional/motivational signs of dependence that mimic the human condition. These signs include increased anxiety-like behavior [39], enhanced sensitivity to CRF type I receptor (CRF_1_R) antagonists [40], [41], blunted function of the neuroendocrine stress system [26], blunted function of the reward system [42], relapse to binge drinking when alcohol is made available (BALs≥0.08 g/dL [26], [30]), and increased willingness to work for access to alcohol [43]. The first withdrawal operant test occurred 10 days into intermittent alcohol vapor exposure. On day 10 of vapor exposure, all rats exhibited increases in lever pressing for alcohol relative to baseline, but this increase was much greater in animals that self-administered sweetened water during adolescence (% increase in g/kg alcohol self-administration relative to the average of last three days of baseline: 147%±46 for Control NonDep; 248%±47 for Control Dep, 113%±46 for Binge NonDep, 114%±38 for Binge Dep; F(1,29) = 3.45, p = 0.07). All animals had a complex memory of the reward lever at this point in the study, having pressed for sweetened water or sweetened alcohol as adolescents, as well as different combinations of sweeteners and alcohol as adults. The trend of a greater increase in responding by controls relative to juvenile binge animals on the 1st day of lever access following a period without access may reflect a transient recovery of the association between the reward lever and sweetened water. Because of this, dependence-induced elevations in drinking were analyzed from two weeks of vapor treatment onward (17–33 days) based on previous data showing that this amount of exposure is sufficient to significantly increase drinking levels [29]. Dependence elicited a significant elevation in alcohol self-administration overall (main effect of vapor condition, F(1,173) = 18.24, #, p<0.01, **Fig. 5**, bar graph). Furthermore, animals with a history of adolescent binge drinking consumed more alcohol as adults during intermittent self-administration testing (main effect of adolescent binge condition, F(1,173) = 5.12, #, p = 0.02, **Fig. 5**). The adolescent treatment×adult treatment interaction was not significant (p = 0.34); however, an *a priori* planned comparison between the two dependent groups indicated that the percent increase from baseline was higher in the Binge Dep group compared to the Control Dep group (with Bonferroni correction, p = 0.02). Binge NonDep and Control NonDep rats did not differ in this measure (p = 0.33).

### Effect of adolescent binge drinking on anxiety in adulthood

**Figure 6:**
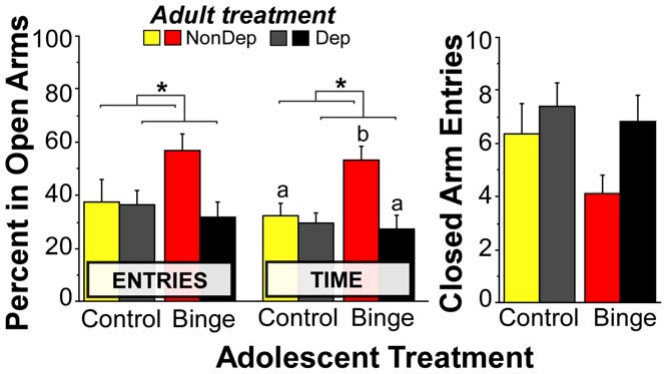
Voluntary binge drinking early in adolescence decreases anxiety-like behavior on the elevated plus maze in non-dependent male Wistar rats. A history of adolescent binge alcohol drinking produced a decrease in anxiety-like behavior (increased percent of time spent in the open arms) that was reversed by alcohol dependence. Data are expressed as mean ± SEM (n = 7–10/group). *p<0.05 main effect of adolescent alcohol binge drinking; ^a,b^p<0.02, with different letters indicating individual group differences (i.e., Binge NonDep is different from BingeDep and Control NonDep).

To test the hypothesis that adolescent binge drinking had long-lasting effects on passive anxiety-like behavior, rats were tested on the elevated plus-maze (EPM) one month after removal from intermittent alcohol vapors (or control air). **Figure 6** illustrates the percent of entries in open arms, percent time spent in open arms, and locomotor activity (closed arm entries) in non-dependent rats with (Binge NonDep) or without (Control NonDep) a history of adolescent binge drinking and alcohol-dependent rats with (Binge Dep) or without (Control Dep) a history of adolescent binge drinking. There was a significant interaction of binge history and alcohol dependence on the percent of time spent on the open arms (F(1,29) = 4.25, p<0.05). *Post-hoc* analyses indicated that non-dependent animals with a history of alcohol binge self-administration exhibited a significant increase in time exploring the open arms relative to non-dependent animals without binge history (p = 0.02, *, **Fig. 6**), signifying binge drinking during early adolescence and moderate drinking in adulthood leads to long term *reductions* in anxiety-like behavior and/or increases in impulsivity [44]–[47]. Subsequent induction of dependence reversed the effects of adolescent binge drinking on anxiety-like behavior in adulthood (*a priori* planned comparison with Bonferroni correction, p = 0.005). Dependence effects were not observed in animals without a previous history of alcohol exposure during early adolescent development (control animals) at this protracted abstinence time point (p>0.05). Closed arm entries were not significantly different among the groups (p>0.05, **Fig. 6**).

### Effect of adolescent binge drinking and alcohol dependence on CRF cells in CeA

**Figure 7:**
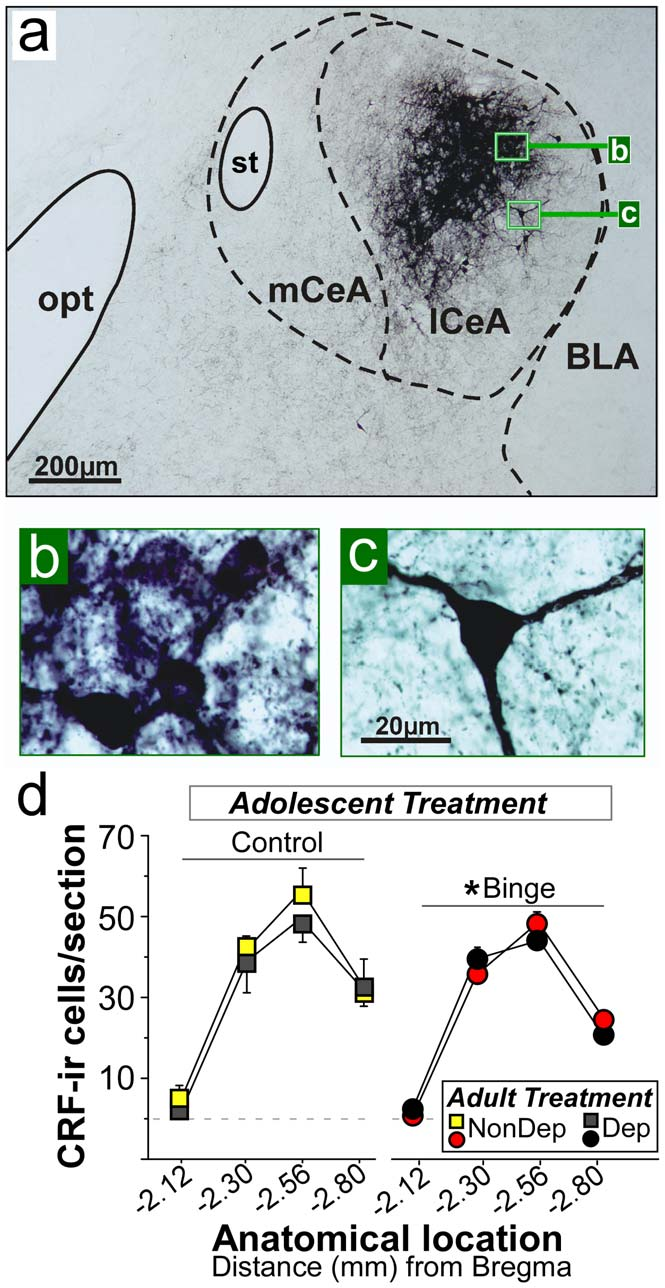
Voluntary binge drinking early in adolescence reduces the number of CRF-ir cells in the lateral division of the central amygdala (lCeA) one month into abstinence from vapors in adult dependent and non-dependent male Wistar rats. Photomicrograph of CRF immunoreactive (CRF-ir) cells and fibers in the lateral portion of the CeA (−2.56 mm posterior to Bregma) in a Control NonDep male rat (a) and high magnification photomicrographs of CRF neurons with dark (b) and light (c) immunolabeling (magnification indicated by bars within the photomicrographs). (d) Shows CRF-ir cell counts (expressed as cells/section) in lateral CeA at various anterior-posterior distances from Bregma in abstinent adult male rats with (Binge) or without (Control) a previous history of binge self-administration in early adolescence and with (Dep) or without (NonDep) chronic intermittent vapor exposure in adulthood. Binge alcohol exposure reduced CRF-ir cell counts in lateral CeA in a manner that was not affected by subsequent chronic intermittent alcohol exposure. Data are expressed as mean ± SEM (n = 4–9/group). *p<0.05 main effect of adolescent alcohol binge drinking. *Abbreviations:* BLA, basolateral amygdala; lCeA, lateral division of the central amygdala, mCeA, medial division of the central amygdala; opt, optic tract.

**Fig. 7** shows photomicrographs of CRF peptide-expressing neurons (**Fig. 7a, b, c**; a NonDep rat without a history of binge alcohol self-administration at −2.56 mm relative to Bregma) and cell counts (**Fig. 7d**) within the lateral CeA. Cells were counted at various anterior-posterior distances from Bregma. Adolescent binge alcohol self-administration produced a significant decrease in CRF-ir cell number (F(1,24) = 5.19, p = 0.03, *, **Fig. 7d**). Alcohol dependence did not exacerbate or rescue the effect of binge alcohol history on CRF-ir cells (p>0.05) or affect CRF-ir cells in animals without binge history at the protracted abstinence time point examined here (p>0.05).

## Discussion

The primary objectives of this series of experiments were to develop an adolescent binge alcohol-drinking model in rodents and to empirically test how binge alcohol exposure during adolescence affects addiction risk in adulthood. This research was motivated by a large epidemiological literature suggesting a strong link between these two factors in humans [5]–[15]. A few key findings emerged from this work. First, involuntary binge-like exposure to alcohol may be aversive, as alcohol injections during early adolescence caused significant and long-lasting *reductions* in drinking of sweetened and unsweetened alcohol in adulthood. Second, genetically heterogeneous strains such as Wistar rats do not spontaneously drink large quantities of alcohol in traditional self-administration procedures, but the present voluntary operant binge-drinking model rapidly initiated high levels of alcohol consumption during early adolescence in the presence of *ad libitum* food and water. All animals reached binge levels (as defined by NIAAA) and cumulative intake averaged 50–60 g/kg within a two-week period. Third, binge alcohol self-administration during early adolescence caused prolonged changes in behavioral and neural measures in adulthood. Adolescent binge drinking significantly increased alcohol drinking in alcohol-dependent and non-dependent adult rats during intermittent testing. Interestingly, Binge NonDep animals exhibited an unexpected high level of exploration (∼60%) into the open arms of the EPM compared to Control NonDep and Binge Dep animals (presumably reflecting either *reduced* passive anxiety-like behavior or increased impulsivity [44]–[47]). Subsequent analysis of brains indicated that, several months following end of adolescence, binge animals had fewer CRF immunolabeled cells in the lateral CeA compared to Control animals. Alterations in the CeA CRF system were specific to adolescent alcohol exposure (dependence in adulthood did not affect the number of CRF cells in lateral CeA one month following end of alcohol vapor). Overall, the present findings suggest that binge drinking during early adolescence impacts alcohol consumption during adulthood in a complex way. Further work using voluntary binge alcohol drinking in adolescent animals will clarify how alcohol-induced neural and behavioral changes functionally relate to vulnerability to addiction and other stress-related disorders in adulthood.

Our data indicate that animals intermittently and involuntarily injected with bolus alcohol during adolescence engaged in the lowest level of intake of rewarding solutions in two-bottle choice tests in adulthood. One possible explanation for this effect is that involuntary alcohol exposure during adolescence was aversive and produced a conditioned taste aversion for the glucose/saccharin mixture paired with those injections. The alcohol-injected group consumed significantly less sweetened water compared to other groups several weeks following adolescent alcohol exposure. They also consumed less sweetened alcohol, suggesting a persistent taste aversion for the solution paired with alcohol injections during adolescence. This interpretation is supported by earlier reports showing bolus alcohol injections produce robust conditioned taste aversions for sweet solutions in rats [48]. Finally, involuntary exposure to binge-like alcohol during early adolescence resulted in long-lasting reductions in *unsweetened* alcohol drinking in adulthood. Because adult alcohol drinking was not altered in animals either consuming sweetened alcohol voluntarily or injected with saline during adolescence, the effects observed in alcohol-injected animals may be attributable to the fact that their first experience with alcohol was involuntary. Involuntary administration of drugs can be aversive, especially if “binge-like” doses (or higher) are administered *before* animals have self-administered the drug themselves, resulting in decreased self-administration ([49], [50], present data). The present data highlight the importance of considering the method of alcohol delivery and its impact on drinking behavior. For example, neural changes in alcohol-injected rats might reflect *decreased* rather than increased addiction vulnerability. Our research ultimately aims to understand how *voluntary* binge alcohol drinking affects the developing brain and risk for addiction later in life; therefore, we developed an operant binge alcohol self-administration model in adolescent rats.

In the adolescent operant binge self-administration model, rats had access to sweetened alcohol in spurts, which elicited voluntary episodic alcohol intake, blood-alcohol levels exceeding 0.08 g% in a number of sessions, and high cumulative alcohol intake during early adolescence. The control group was exposed to the same operant conditions as the binge group except only sweetened water was self-administered (lever presses were capped to be equivalent to the amount of sweetener consumed by the binge group). This allowed us to directly assess the specific effects of adolescent alcohol exposure on brain and behavioral measures in adulthood. One limitation of the present study is that we do not know how adolescent exposure to sweetened water specifically affects drinking in adulthood. A recent report showed that sugar intake during adolescence is protective against alcohol drinking later in adulthood [51]. Yet, as the present study shows, when sugar is combined with alcohol, it promotes binge-like alcohol drinking and high cumulative intake in young animals. Thus, the suppressive effect of adolescent sugar exposure on alcohol drinking in adulthood appears to be overshadowed by the facilitatory effect of adolescent alcohol exposure on alcohol drinking in adulthood.

Adolescent binge drinking had prolonged effects on drinking behavior in adulthood, but the alterations were only evident under certain testing conditions. Binge and control rats had similar drinking behavior during daily baseline testing (before chronic vapor/air exposure) in adulthood. However, when animals were tested every 3–7 days during the vapor phase of the experiment, a history of adolescent binge alcohol drinking produced a moderate, but significant increase in adult alcohol responding. This augmentation occurred regardless of vapor condition suggesting that adolescent binge drinking exacerbates the *relapse* to drinking that occurs after a few days without access to alcohol self-administration in non-dependent and dependent animals [30]. Past studies have shown that intermittent operant alcohol testing in non-dependent rats produces increases in alcohol drinking that are sensitive to pharmacological manipulation of brain stress systems [52], [53]. This effect is reminiscent of the alcohol deprivation effect, which is a well-established phenomenon wherein rats exhibit transient increases in alcohol consumption following a period of alcohol deprivation [54]–[56].

Chronic exposure to alcohol intoxication/withdrawal cycles (dependence induction) increased alcohol drinking in all rats 6–8 hours into withdrawal from vapors, as previously reported [26], [30], [43]. The effect of binge on relapse-like drinking may have been slightly enhanced in alcohol-dependent rats relative to non-dependent controls, although this result was not statistically significant (a main effect of adolescent treatment and an *a priori* planned comparison between the control and binge dependent groups were significant, but the adolescent treatment×adult treatment interaction was not). One of the challenges with the operant binge model is dealing with reduced statistical power because of increased variability in the experimental treatment (e.g., adolescent alcohol is delivered via self-administration). Consequently, larger sample sizes may be required to detect significant interaction effects of adolescent and adult alcohol histories on alcohol drinking behavior in genetically heterogeneous rats. Regardless, the present study showed that adolescent binge alcohol drinking affects relapse-like drinking in adulthood. Previous studies suggest that alcohol drinking during adolescence does not affect baseline (consistent with our results) or deprivation-induced/relapse drinking during adulthood (not consistent with our results), but does render rats more sensitive to stress-induced increases in alcohol drinking during adulthood ([57], [58], not measured in the present study). The pattern and level of voluntary alcohol exposure (i.e., binge drinking vs. non-binge drinking) may have contributed to the observed changes in relapse-like drinking in adulthood in the present study. Prior alcohol experience (e.g., repeated withdrawals) and stress exhibit additive and substitutive effects on alcohol-related behaviors (e.g., self-administration and anxiety-like behavior; [59], [60]), and the magnitude of these effects depends on the age of the animal [61]. Together, these results suggest that rats with a history of adolescent binge alcohol drinking may be more susceptible to the facilitatory effects of acute stress on relapse to heavy drinking after a period of abstinence.

Adolescent binge drinking had long-lasting (i.e., several months) effects on exploratory behavior on the elevated plus maze (EPM). Early binge drinking elicited an unexpected high level of exploration onto the open arms if animals had limited exposure to alcohol in adulthood (i.e., non-dependent drinking). Binge NonDep rats spent 60% of their time exploring the open arms, suggesting either *decreased* passive anxiety-like behavior or increased impulsivity [44]–[47] in these animals. Subsequent exposure to repeated daily cycles of high-dose alcohol vapor inhalation and withdrawal (i.e., dependence) significantly reduced open arm exploration in Binge Dep rats relative to the non-dependent comparison group (Binge NonDep rats). There was no effect of dependence on anxiety-like behavior in animals without a history of adolescent binge drinking (Control NonDep and Control Dep rats exhibited similar behavior on the EPM) at this protracted abstinence time point (∼1 month). Open arm exploration in Binge Dep, Control Dep, and Control NonDep rats was ∼40%, which is similar to the levels observed one month into abstinence from alcohol in previous studies ([62], but see [59]). Weeks or months into abstinence, an external stressor [62], [63] or stimulation of CRF/CRF type I receptor system [60], [61], [63], [64] is usually necessary to reveal the long-term effects of alcohol dependence induction on the behavioral stress system. Strict interpretation of the interaction between adolescent and adult treatment in the present study would suggest that adolescent binge drinking increases sensitivity to chronic alcohol vapor exposure on open arm exploration in the EPM, but the difference in EPM behavior in the two binge groups appears to be attributable to an exceptionally high level of open arm exploration in the NonDep Binge rats rather than a low level of open arm exploration in the Dep Binge rats.

CRF immunolabeled cells were counted in the lateral division of the CeA. The lateral and medial divisions of the CeA differ in terms of their neuropeptide content, origin of incoming afferents, and target sites of efferent projections (reviewed in [65]). Relative to the medial CeA, the lateral CeA contains a much higher density of neuropeptides such as CRF [66]–[69]. The medial CeA receives prominent inputs from the lateral CeA and other amygdaloid nuclei and sends dense projections to effector regions such as hypothalamus and brainstem nuclei [65]. Medial CeA projection neurons receive excitatory inputs from BLA as well as inhibitory inputs from lateral CeA and intercalated GABA cells, although it is not yet known precisely which synaptic connections govern emotion- and alcohol-related behavior [70], [71]. In a series of slice electrophysiology experiments, it has been shown that alcohol, as well as pro-anxiety and anti-anxiety neuropeptides, modulate GABAergic transmission in the medial CeA of rats, and these effects are often up-regulated following the transition to alcohol dependence [52]. More generally, the amygdala receives strong inputs about emotionally relevant stimuli in the external environment and internal milieu, and communicates between nuclei and also within the CeA to convert sensory information into appropriate behavioral and physiological responses.

Adolescent binge alcohol drinking produced decreases in CRF-labeled cells in lateral CeA that were evident months following end of adolescence. A recent study showed that consumption of alcohol-containing liquid diet produced decreases in CRF-immunoreactive content (cells were not individually counted) in the CeA of adolescent rats, but not in adults [61] shortly after alcohol liquid diet treatment ended at 45 days of age. This change in immunoreactivity was interpreted to be reflective of increased release of CRF. In the present study, however, alterations in the CRF population within the CeA were evident long after the adolescent exposure occurred (several months after the initial two-week long binge drinking period). Together the studies suggest CRF cells in the CeA may be particularly sensitive to moderate-to-high alcohol exposure during adolescent development.

A reduction in peptidergic immunolabeled cells and fibers may be interpreted in different ways, depending on the experimental procedure used to label those cells. Subthreshold concentrations of antiserum have been used as an approach to decrease immunolabeling (i.e., detectability) of cells with depleted peptide store [72]–[75]. Under these experimental conditions, highly active neurons are continually releasing peptide, and consequently peptide stores within the soma fall below the level of detectability. Thus, increased cellular activity in a brain region results in a lower (not higher) number of cells detected by sub-threshold immunolabeling [72]–[75]. In the present study, we intentionally used an antiserum with high specificity for CRF and optimal staining conditions (including ideal post-fixation time, concentration of antiserum, and nickel enhancement) to produce a strong signal in CRF-producing neurons with even low peptide content. In addition, a more detailed analysis of CRF immunoreactivity indicated reduced CRF-ir cell number came primarily from a loss of darkly stained cells (containing high stores of peptide) rather than CRF cells with medium/light immunoreactivity (within which peptide release would more likely result in peptide stores low enough to fall below detectability, data not shown). This suggests that the reduction in CRF-ir cell number may reflect a long-term loss in CRF-producing cells in the lateral CeA following adolescent binge drinking. Future studies utilizing alternate cell-labeling procedures (e.g., in situ hybridization to measure CRF mRNA) could provide evidence for or against this interpretation.

It is difficult to draw meaningful conclusions from the present data about the relationship between amygdalar CRF cell number, alcohol drinking, and anxiety-like behavior. However, an abundance of data suggests that alterations in amygdalar CRF relate to alcohol drinking and anxiety-like behavior in rodents, particularly those with a history of chronic high-dose alcohol exposure. Alcohol-preferring rats exhibit high levels of anxiety-like behavior and excessive drinking [76], and have lower CRF mRNA expression and peptide content, but exhibit higher sensitivity to CRF administration in the CeA relative to non-preferring rats [76], [77]. CRF immunoreactivity is reduced in alcohol-dependent rats two hours into withdrawal from chronic intermittent vapors, a time when these animals engage in heavy alcohol consumption (interpreted as increased CRF release [35]). Slice electrophysiology experiments suggest that alcohol dependent rats are more sensitive to the facilitatory effects of CRF on GABAergic transmission in CeA during acute withdrawal compared to alcohol naïve rats, which is paralleled by increased CRF mRNA in CeA at the same time point [53]. Rats made dependent on alcohol via liquid diet exhibit increased CRF-like immunoreactivity in amygdala six (but not three) weeks into abstinence from alcohol liquid diet [37]. Finally, CRF knockout mice consume significantly more alcohol than wild-type mice [78]. It is not known whether the complex relationships between changes in CRF peptide expression in the amygdala, heavy drinking, and anxiety are corollary or causal in nature. The functional connection between reduced CRF peptide expression in CeA and behavioral effects observed in animals with adolescent binge alcohol history is a topic that warrants further investigation.

In summary, the current study showed that intermittent bouts of moderate-to-high voluntary alcohol drinking early in adolescence (postnatal days 28–42) had long-lasting effects on addiction-related behaviors and brain circuitry in male rats. An important direction of future work should be to determine whether there are sex differences in the behavioral and neural consequences of adolescent binge drinking, as binge drinking is higher in adolescent females (Karanikas and Richardson, unpublished findings). At this point it is also unknown whether the neural and behavioral effects observed in males were dependent on the pattern (i.e., episodic drinking) and/or level (i.e., blood alcohol levels reaching 0.08 g% and >50 g/kg total intake) of exposure to alcohol. However, an important advantage of the operant model is that such variables can be manipulated to determine the specific behavioral characteristics and exposure thresholds that impact the developing brain during adolescence to alter mental health risks in adulthood. Thus, the voluntary adolescent binge model described here is expected to be a useful tool for identifying the neurobiological mechanisms underlying links between early episodes of heavy drinking and neurological and behavioral deficits later in life.

## Materials and Methods

### Ethics statement

All procedures adhered to the National Institutes of Health *Guide for the Care and Use of Laboratory Animals* and were approved by the Institutional Animal Care and Use Committee. All efforts were made to reduce the number of animals used and to minimize pain and suffering.

### Animals

Sixty adolescent male Wistar rats obtained from Charles River (Kingston, NY) were used in this study. Animals were 21 days old upon arrival and weighed between 52–72 g (mean body weight = 62.08±0.85 g) at the start of the experiment. Animals were single-housed in the involuntary binge alcohol experiment and group-housed (2–3 rats/cage) in the voluntary binge alcohol drinking experiments. Animals were housed in standard plastic cages with wood chip bedding under a 12 hour light/12 hour dark cycle (lights off at 8 AM) and were given *ad libitum* access to food and water. All experiments were conducted in the dark cycle.

### Effect of involuntary vs voluntary alcohol exposure during early adolescence on non-dependent drinking in adulthood

To develop a binge alcohol model, we first compared the effects of involuntary vs. voluntary exposure to alcohol during adolescence on drinking in adulthood. Adolescent male Wistar rats were allowed four 30-min bouts of access to a single bottle of sweetened alcohol on each of five separate days during adolescence. Adolescent drinking took place in the home cage in the absence of food and water on postnatal days 27, 30, 33, 36, and 39. Three other groups were allowed to drink sweetened water in parallel with the first group. Immediately after the fourth bout on each treatment day, rats were injected with either 2.0 g/kg alcohol (alcohol injected) an equivalent volume of saline (control injected) or nothing (control drinking).

Sweetened alcohol was made up of 5% w/v ethanol plus 3% glucose/0.125% saccharin in tap water. In the 60-min breaks between each bout the experimental bottles were removed from the cage, and food and water were available. Several weeks later in adulthood (∼postnatal day 72), rats were allowed to drink the following during daily 30-min sessions (1 session per day): 1) sweetened water (3% glucose/0.125% saccharin, first three days of testing), 2) sweetened alcohol (3% glucose/0.125% saccharin/10% w/v alcohol for the first two days of testing followed by 0.125% saccharin/10% w/v alcohol for the next two days), 3) and unsweetened 10% w/v alcohol (last 13 days of testing).

#### Measurement of blood alcohol levels

Plasma (5 µl) collected by cutting the tip of the tail was used for measurement of blood alcohol levels using an Analox AM 1 analyzer (Analox Instruments, Lunenburg, MA). The reaction is based on the oxidation of alcohol by alcohol oxidase in the presence of molecular oxygen (alcohol+O_2_→acetaldehyde+H_2_O_2_). The rate of oxygen consumption is directly proportional to the alcohol concentration. Single-point calibrations were done for each set of samples with reagents provided by Analox Instruments (0.025–0.400 g% or 5.4–87.0 mM).

### Voluntary (self-administered) binge alcohol drinking experiments

#### Operant Boxes

The operant boxes (Coulbourn Instruments, Allentown, PA) utilized in the present study had two retractable levers located 4 cm above a grid floor and 4.5 cm to either side of a two-well acrylic drinking cup. Operant responses and resultant fluid deliveries were recorded by custom software running on a PC computer. A single lever-press activated a 15 rpm Razel syringe pump (Stanford, CT) that delivered 0.1 ml of fluid to the appropriate well over a period of 0.5 sec. Lever presses that occurred during the 0.5 sec of pump activation were not recorded and did not result in fluid delivery. Operant boxes were individually housed in sound-attenuated ventilated cubicles to minimize environmental disturbances. The right lever always produced a delivery of experimental solution and the left lever, when available, always produced a water delivery.

#### Adolescent model of binge self-administration: Training (postnatal days 25–27)

The adolescent binge-drinking period was 14 days long, beginning on postnatal day 28 and ending on postnatal day 42. During the three days prior to the start of the treatment period (postnatal 25–27) all animals were trained to self-administer sweetened water. On the first training day (postnatal day 25), Wistar rats were placed in operant boxes for the first overnight session at the start of the dark cycle. Rats were placed in operant boxes in pairs for 12 hours (dark cycle); a single lever was available for 12 consecutive hours and presses on that lever resulted in delivery of 0.1 ml sweetened solution (3% glucose, 0.125% saccharin, and tap water). On the second training day (postnatal day 26), all rats were again placed in operant boxes, this time single-housed, and allowed 12 consecutive hours of access to a single lever that, when pressed, resulted in access to 0.1 ml of sweetened solution. The third training day (postnatal day 27) was similar to the previous day with a single exception: rats were introduced to the “binge” schedule of lever access. In the binge schedule, rats were allowed access to a single lever in six separate 30-min mini-sessions distributed across the 12-hour operant session, and separated by 90-min periods with no lever access. During the training period (postnatal days 25–27), rats were given *ad libitum* food access but no water was available during operant sessions. During the adolescent binge exposure period (postnatal days 28–42), food and water were available to the rats *ad libitum*.

#### Adolescent model of binge self-administration: Binge drinking (postnatal days 28–42)

On postnatal day 28, rats were divided into two groups matched for operant responding for sweetened solution: one group (alcohol binge group) would be allowed 14 days of 12-hour operant sessions (six 30-min mini-sessions per 12-hour period) in which lever presses would result in delivery of 0.1 ml of a solution that contained sweetened 10% (w/v) alcohol, and the second group (sweetened water control group) would be allowed 14 more days (postnatal days 29–42) of 12-hour operant sessions in which lever presses would result in delivery of 0.1 ml sweetened water solution. Beginning with the sixth of those binge sessions, operant responding by sweetened water control rats was capped such that rats were only allowed eight lever presses in each of the six mini-sessions during the 12-hour operant session; the purpose of this cap was an attempt to match rats in the two groups for operant responding history. Water and food were always available *ad libitum* during and following operant sessions from postnatal day 28 onward. On postnatal day 43, rats were once again placed in operant self-administration boxes for binge sessions, except that alcohol binge rats were immediately removed from boxes following mini-sessions (“bouts”) during which they exhibited high alcohol responding (levels anticipated to produce BALs = 0.08 g%), bloods collected, and rats returned to the home cage. For each alcohol binge rat that was removed from the operant box, a single control rat was also removed from its operant box and blood collected in parallel to control for the effects the blood sampling procedure on subsequent measures in adulthood. Following blood collection, all rats were returned to the home cage and, with the exception of body weight measurements, left undisturbed (one month period with no access to alcohol or sweetened water before behavioral testing began in adulthood, ∼postnatal day 78). Adult rats were then tested for spontaneous operant alcohol self-administration (baseline, non-dependent drinking), dependence-induced alcohol self-administration during acute withdrawal from chronic intermittent vapors (Control Dep and Binge Dep rats; air control exposure was used for Control NonDep and Binge NonDep rats), and post-dependent anxiety-like behavior (measured after one month after removal from vapors), as described below.

#### Baseline drinking in adulthood

On postnatal day 78, rats were again placed in operant boxes (4 hours into the dark cycle) for 30-min operant sessions. During 30-min operant sessions conducted in adulthood, all rats were allowed to respond for sweetened water (3% glucose/0.125%saccharin in tap water, 1 day, **Fig. 4a**), sweetened alcohol (2 days of 3% glucose/0.125% saccharin/10% w/v alcohol left graph, **Fig. 4b**; 2 days of 0.125% saccharin/10% w/v alcohol, right graph, **Fig. 4b**), and unsweetened alcohol (10% w/v alcohol, 13 days, **Fig. 4c**) in a concurrent, two-lever, free-choice contingency. Lever-presses were reinforced on a continuous fixed ratio-1 (FR1) schedule such that each response resulted in delivery of 0.1 ml of fluid.

#### Dependence induction in adulthood (intermittent alcohol vapor inhalation)

To induce alcohol dependence, standard rat cages were housed in separate, sealed, clear plastic chambers into which alcohol vapor was intermittently injected. This procedure has been described in detail elsewhere [29], [79]. The target range for BALs in dependent rats during vapor exposure was 150–200 mg% [30]. Non-dependent rats were treated in parallel except they were continuously exposed to ambient air. Vapor was delivered on an intermittent schedule (14 hours on/10 hours off). Typically, rats are exposed to vapor for four weeks before post-vapor testing begins because this schedule of exposure has been shown to induce mild physical dependence [26] and robust emotional/motivational dependence (see [30] for details about the physiological parameters of the model and [27] for a review of the clinical relevance of the model). However, in this study, rats were tested earlier in the vapor exposure protocol to determine drinking behavior during the transition to alcohol dependence [52], [53].

#### Alcohol drinking during the transition to dependence in adulthood

The alcohol dependence model used in these studies reflects the natural progression of alcohol dependence in which alcohol exposure occurs in a series of extended exposures followed by periods of withdrawal [27]. In this model, experimental groups of rats are made dependent on alcohol using chronic intermittent alcohol vapor exposure (e.g., several weeks of daily cycles of intoxication by alcohol vapors followed by withdrawal; physiological parameters are described in [30]). Groups of animals with and without history of binge alcohol self-administration in adolescence were divided into two groups matched for baseline operant self-administration behavior. Those two groups were then exposed to either chronic intermittent (14 hours on/10 hours off) alcohol vapor (dependent group) or ambient air (non-dependent group), starting on postnatal day 130. Dependence-induced drinking was measured 8–10 hours after withdrawal from vapors every 3–7 days, as previously described [26].

#### Elevated Plus Maze (EPM) test of anxiety-like behavior in adulthood

The EPM is a widely used test of anxiety-like behavior [44], which is sensitive to putative anxiogenic and anxiolytic drugs [39], [80], [81]. The methods used here were as previously described [59], [82]. The maze was made of black Plexiglas and consisted of four arms (50 cm long×10 cm wide); two arms had 40 cm high dark walls (closed arms), and two arms had 0.5 cm high ledges (open arms). The floor of the apparatus was 50 cm high. Testing occurred in a dimly lit room where open arms received 1.5 to 2.0 lux of illumination and closed arms received <1 lux of illumination. Animals were tested 4 weeks following termination of alcohol vapor exposure in alcohol-dependent rats and 2–4 hours into the dark cycle. Rats were placed individually onto the center of the maze facing a closed arm and removed after a 5-min test period. Behavior was recorded and scored by one experimenter blind to the treatment condition of the animals. The apparatus was wiped clean with water and dried between subjects. The primary measures were the percent of total arm time and number of entries (defined as all four paws entering an arm) into the open arms (i.e., 100×open arm/(open arm+closed arm)), which are validated indices of anxiety-related behavior or “unprotected exploration” [45]. The number of entries into the closed arms and total arm entries are validated indices of locomotor activity based as documented in factor analysis studies [45], [46]. Thus, we measured these variables to assure that a reduction in open arm entries indicates a reduction in risky exploration (heightened anxiety-like behavior) and not a reduction in locomotor activity overall.

#### Brain tissue collection and immunohistochemistry

Rats were deeply anesthetized with chloral hydrate (35%, 2 ml/kg, a drug that does not affect stress-related immediate early genes or peptides mRNA levels), and intracardially perfused with 4% paraformaldehyde/0.1 M borate buffer, pH 9.5. Brains were post-fixed for 4 hours, submerged in 20% sucrose solution for 24–48 hours at 4°C, snap frozen in isopentane (2-methylbutane, Sigma), and stored at −80°C until sectioning on a microtome into 35 µm coronal sections. Tissue sections were then stored at −20°C in a cryoprotectant solution (50% 0.1 M phosphate buffered saline, 30% ethylene glycol, and 20% glycerol) until immuohistochemistry. Free-floating sections were processed for immunohistochemical labeling of CRF cells and fibers in the CeA. Sections underwent several rinses with 0.1 M PBS and 0.1 M PBS-Triton X-100 (PBS-TX) followed by a 30-min incubation in a 0.3% hydrogen peroxide/PBS solution to block endogenous peroxidase activity. Sections were rinsed several times in PBS-TX and non-specific binding was then blocked with a 60-min incubation in 5% milk/PBS. Sections were rinsed again in PBS-TX, then incubated 18–20 hours 4°C at with 10% normal goat serum in a milk/PBS-TX solution containing primary rabbit anti-h/rCRF antibody (1∶5000, generously donated by Dr. Wylie Vale, The Salk Institute). The following day, sections were rinsed in a series of PBS-TX before a 2-hour incubation at room temperature in biotin-tagged secondary antibody solution (goat anti-rabbit, 1∶200, catalog no. W0117; Vector Laboratories, Burlingame, CA) at 4°C followed by a 60-min incubation at room temperature in avidin-biotin complex solution (1∶200∶1; catalog no. PK-6100, Vector Laboratories). Staining was visualized with NiCl-enhanced, 3,3′-Diaminobenzidine tetrahydrochloride (catalog no. 078K8200, Sigma-Aldrich Co., St Louis, MO). After immunohistochemical labeling, sections were slide mounted, dried overnight, then coverslipped, for microscopic analysis.

#### Microscopic Analysis

CeA CRF-immunoreactive (-ir) cell bodies were counted by an experimenter blind to the conditions of the animals of the study using a Leica microscope and a standard thumb-operated tally counter at 400× magnification (40× objective and 10× eyepiece). The sections analyzed were −2.12 mm, −2.30 mm, −2.56 mm, and −2.80 mm relative to Bregma according to the Rat Brain in Stereotaxic Coordinates Atlas [83], [84]. Many cells within the CeA had dark immunolabeling on the outside of the cell, which appeared to be coming from immunoreactive dendritic or axonal fibers and not dark immunoreactivity within the soma itself. Cells were only counted as CRF-ir if they had immunolabeling throughout the cell body.

#### Statistical Analysis

Simple regression analyses were used to analyze the correlative relationship between g/kg intake and resultant blood alcohol levels in adolescent binge animals. Drinking data (number of responses, g/kg intake, etc.) and body weight (g/kg) were analyzed using a two-factorial multivariate analysis of variance (MANOVAs) with adolescent binge drinking treatment as between- and day as within-subjects variables. Dependence-induced drinking (% increase in g/kg from baseline in 30-min tests 6–8 hours into withdrawal from alcohol vapors for dependent groups and air control for non-dependent groups) was analyzed using a three-factorial MANOVA with adolescent binge drinking treatment and adult dependence treatment as between-subject factors and test day as a within-subjects factor. Behavioral measures on the elevated plus maze were analyzed using two-factorial ANOVAs with adolescent binge drinking treatment and adult dependence treatment as between-subject factors. CRF-ir cell number was analyzed using three-factorial ANOVAs with adolescent binge drinking treatment and adult dependence treatment as between-subject factors and anatomical location relative to bregma as a within-subject factor. Bonferroni corrections were used for *a priori* planned comparisons and *post-hoc* comparisons. Differences were considered significant when p≤0.05. Wherever appropriate, data are expressed as mean ± SEM.
